# Once weekly paclitaxel associated with a fixed dose of oral metronomic cyclophosphamide: a dose-finding phase 1 trial

**DOI:** 10.1186/s12885-018-4678-x

**Published:** 2018-07-31

**Authors:** Diane Pannier, Antoine Adenis, Emilie Bogart, Eric Dansin, Stéphanie Clisant-Delaine, Emilie Decoupigny, Anne Lesoin, Eric Amela, Sandrine Ducornet, Jean-Pierre Meurant, Marie-Cécile Le Deley, Nicolas Penel

**Affiliations:** 1 Medical Oncology Department, Centre Oscar Lambret, 3, rue F Combemale, 59020 Lille Cedex, France; 2 Clinical Research and Innovation Department, Centre Oscar Lambret, Lille, France; 3 INSERM CESP Oncostat Team, Paris-Sud, Paris-Saclay University, Orsay, France; 4 Medical School, Lille-Nord-de-France University, EA2694 Research Unit, Lille, France

**Keywords:** Dose-finding phase 1 trial, Metronomic cyclophosphamide, Weekly paclitaxel

## Abstract

**Background:**

The primary aim of this trial was to determine the recommended phase II dose (RP2D) of weekly paclitaxel (wP) administered in combination with oral metronomic cyclophosphamide (OMC).

**Methods:**

Patients ≥ 18 years of age with refractory metastatic cancers were eligible if no standard curative measures existed. Paclitaxel was administered IV weekly (D1, D8, D15; D1 = D28) in combination with a fixed dose of OMC (50 mg twice a day). A 3 + 3 design was used for dose escalation of wP (40 to 75 mg/m^2^) followed by an expansion cohort at RP2D. Dose-limiting toxicity (DLT) was defined over the first 28-day cycle as grade ≥ 3 non-hematological or grade 4 hematological toxicity (NCI-CTCAE v4.0) or any toxicity leading to a dose reduction.

**Results:**

In total, 28 pts. (18 in dose-escalation phase and 10 in expansion cohort) were included, and 16/18 pts. enrolled in the dose-escalation phase were evaluable for DLT. DLT occurred in 0/3, 1/6 (neuropathy), 0/3 and 2/4 pts. (hematological toxicity) at doses of 40, 60, 70 and 75 mg/m^2^ of wP, respectively. The RP2D of wP was 70 mg/m^2^; 1/10 patients in the expansion phase had a hematological DLT. At RP2D (*n* = 14), the maximal grade of drug-related adverse event was Gr1 in three patients, Gr2 in six patients, Gr3 in one patient and Gr4 in one patient (no AE in three patients). At RP2D, a partial response was observed in one patient with lung adenocarcinoma.

**Conclusion:**

The combination of OMC and wP resulted in an acceptable safety profile, warranting further clinical evaluation.

**Trial registration:**

TRN: NCT01374620; date of registration: 16 June 2011.

**Electronic supplementary material:**

The online version of this article (10.1186/s12885-018-4678-x) contains supplementary material, which is available to authorized users.

## Background

Metronomic chemotherapy refers to the frequent, typically daily, administration of cytotoxic drugs at doses that are significantly lower than the maximum-tolerated dose, with no prolonged drug-free breaks. Oral cyclophosphamide-based metronomic chemotherapy (OMC) is the most largely studied metronomic regimen, with greater than 30 retrospective studies and phase II trials reporting in vivo anti-angiogenic and immune-modulatory properties and significant clinical anti-tumor activity, which has been confirmed in heavily treated patients who have exhausted all effective treatments [1–3].

The mode of action of paclitaxel involves the stabilization of microtubules through the inhibition of the depolymerization process [4, 5]. This inhibition of de-polymerization is observed during the metaphase/anaphase transition of mitosis [5]. Paclitaxel exhibits a wide spectrum of anti-tumor activity, including breast cancers, even those refractory to anthracyclines; lung cancers; squamous cell carcinomas of the upper respiratory/digestive tracts; stem cell tumors; lymphomas; and Kaposi tumors [6–14].

Compared with 3-week cycles, weekly administration of paclitaxel induces a clear increase in dose-intensity without significant enhancement of toxicity for fragile or heavily pretreated patients with ovarian [8, 9], lung [10, 12] gastric cancers [11] or bladder cancer [15] . However, the clinical benefit had to be weighted in regards of the inconvenience of returning to clinic weekly for administration of the drug. Because of its manageable toxicity profile, weekly administration of paclitaxel remains in everyday practice a largely used as palliative chemotherapy, especially in ovarian and bladder cancer patients [8, 9, 15]. Weekly paclitaxel is one of the comparator arm in recent randomized phase III trial comparing the activity of atezolizumab versus chemotherapy in advanced bladder cancer (IMVigor211 Trial, NCT02302807). In the IMVigor211 trial, atezolizumab failed to demonstrate superiority compared to classical chemotherapy, and weekly paclitaxel appears the most effective drug.

We hypothesize that metronomic cyclophosphamide and weekly paclitaxel combination is feasible combination. In this context, we performed a multi-center dose-finding phase I trial to determine the recommended phase II dose of weekly paclitaxel administered in combination with metronomic cyclophosphamide and to evaluate the safety and preliminary signs of activity of this combination.

## Methods

### Study design

This was a 3 + 3 dose-escalation single-center study. The primary objective was to determine the recommended phase II dose of weekly paclitaxel administered in combination with a fixed dose of OMC.

### Patients

The main inclusion criteria were histology-proven malignancy, patients having exhausted all available standard of care, documented disease progression at study entry, target measurable according to RECIST 1.1, wash-out period of 28 days after the prior treatment, no persistent toxicity related to prior therapies, age between 18 and 65 years, WHO performance status ≤2 within 7 days prior to the study entry, correct biological parameters (Absolute granulocytes ≥1500/mm^3^, platelets ≥100,000/mm^3^, hemoglobin ≥9 g/L, albuminemia ≥36 g/L, lymphocytes count ≥700/mm^3^, bilirubin and AST/ALT ≤3 ULN or ≤ 5 ULN in case of liver metastasis, and creatinine clearance ≤60 mL/min), negative pregnancy test within 7 days, use of effective contraceptive measures, and absence of any psychological, familial, sociological or geographical condition potentially hampering compliance with the study protocol and follow-up schedule and before registration. Written informed consent must be provided according to ICG/GCP and national regulations. Exclusion criteria were as follows patients undergoing simultaneous therapy with other anticancer agents, prior treatment with paclitaxel, brain or leptomeningeal metastasis, patients not able to swallow and absorb the oral investigational agent, prior symptomatic neuropathy, uncontrolled infection and contraindication to metronomic cyclophosphamide (urinary tract infection, prior hemorrhagic cystitis, and insipid diabetes).

### Dose-escalation process and definition of the dose-limiting toxicity

In every dose-levels, cyclophosphamide dose was 50 mg twice a day. We have already designed two prior clinical trials based on 50 mg cyclophosphamide twice a day as backbone of metronomic chemotherapy regimen [2, 3]. The safety profile was favorable and allows furtther clinical investigations, including in heavily pretreated patients.

Eligible patients received weekly paclitaxel. Seven dose-levels were planned: 40 mg/m^2^, 60 mg/m^2^, 70 mg/m^2^, 75 mg/m^2^, 80 mg/m^2^, 85 mg/m^2^ and 90 mg/m^2^. Paclitaxel was administered days 1, 8 and 15 of 28-day cycles via a 60-min infusion on an outpatient basis. Patients received intravenous pre-medication, including 8 mg dexamethasone, 200 mg cimetidine and 5 mg dexchlorpheniramine. Standard anti-emetics (mainly metoclopramide, 10 mg) were prescribed as clinically indicated by the treating physician. Oral metronomic cyclophosphamide was administered continuously at 50 mg twice a day. Paclitaxel was administered if all the following criteria were met: performance status ≤2, hemoglobin ≥9 g/L, granulocytes ≥1500/mm^3^, platelets ≥100,000/mm^3^, AST/ALT and bilirubin < 3 ULN and absence of dose-limiting toxicities (DLT).

DLTs were weekly assessed during the first 28 days of treatment and included the following toxic events (NCI-CTCAE v4.0): prolonged (> 7 days) grade 4 neutropenia, febrile neutropenia with fever ≥38.5 °C, grade 4 thrombocytopenia, hemorrhage related to thrombocytopenia, hematological toxicity not allowing paclitaxel administration on Days 8 or 15, grade 3 or 4 non-hematological toxicity and oral metronomic interruption for at least 4 days.

We planned an expansion cohort of 10 additional patients at the dose identified as the recommended phase II dose to better explore the tolerability and the activity of this combination.

### Other objectives

Other objectives were to describe the nature and severity of adverse events (NCI-CTCAE v4.0), assess the response after 2 cycles according to RECIST 1.1, estimate the progression-free and overall survival from the date of inclusion, and estimate the growth modulation index (GMI, defined as the ratio between time to progression on study treatment and time to progression on prior treatment). We have described distribution of adverse events in the 1st cycle of treatment as well as the distribution of adverse events observed during the overall treatment.

### Statistical considerations

All estimates were provided with their 95% confidence intervals (95%CI). Progression-free and overall survival curves were estimated using the Kaplan-Meier method. Analyses were performed using Stata/SE (version 13.1) statistical software (StataCorp LP, College Station, TX, USA).

### Ethical considerations

This study was approved by the regional Ethics Committee (“Comité de Protection des Patients Nord-Ouest III”, date of approval: 02 March 2011) and the French Health Products Safety Agency (“Agence Française de Sécurité Sanitaire et des Produits de Santé”, Date of 13 May 2011). This study was registered in the ClinicalTrial.gov Register (NCT01374620). Written informed consent was obtained from each patient.

## Results

### Description of the population

Twenty-eight patients were included between June 2011 and February 2013: 19 men (68%) and 9 women (32%). The median age was 54.5 years (range, 26–67). The primary lesions were colorectal adenocarcinomas (*n* = 9, 32%), soft tissue sarcomas (*n* = 4, 14%), head and neck carcinoma (*n* = 3, 11%), other digestive carcinomas, liver cancer, lung cancer, (2 each, 7%), renal cell carcinoma, cervical cancer, bone sarcoma, testis cancer, ocular melanoma and unknown primary site (1 each, 4%). Twenty-seven patients (96%) had metastatic disease, mainly involving the lung (*n* = 20, 71%), liver (*n* = 10, 36%) or lymph nodes (*n* = 11, 39%). At study entry, the performance status was PS = 0 in 19 patients (68%), PS = 1 in 8 patients (29%) and PS = 2 in 1 patient (4%). Previous treatments included surgery in 24 cases (86%), radiotherapy in 15 cases (54%) and previous systemic chemotherapy or targeted treatment in 27 cases (96%). The number of prior systemic treatment lines was 0 in 1 case (4%), one in 3 cases (11%), two in 2 cases (7%), and 3 or more in 22 cases (78%). Only one patient previously received cyclophosphamide (cyclophosphamide-vinorelbine for a para-testicular rhabdomyosarcoma), and no patient received prior paclitaxel.

### Dose escalation (Table 1)

Three patients were enrolled at dose-level 1 (40 mg/m^2^ of weekly paclitaxel), seven patients at dose-level 2 (60 mg/m^2^), 14 patients (including four patients for dose escalation and 10 patients in the expansion cohort) at dose-level 3 (70 mg/m^2^) and four patients at dose-level 4 (75 mg/m^2^). All patients received at least one dose of paclitaxel.

**Table 1:** Summary of dose escalation

Dose-level	Number of patients enrolled	Number of patients evaluable for DLT	Number of patients with DLT	Details regarding the observed DLTs
1 (40 mg/m^2^)	3	3	0	–
2 (60 mg/m^2^)	7	6	1	Peripheral neuropathy
3 (70 mg/m^2^)	4	3	0	–
4 (75 mg/m^2^)	4	4	2	Febrile neutropenia
				Leucopenia not allowing paclitaxel administration
Expansion (70 mg/m^2^)	10	10	1	Leucopenia not allowing paclitaxel administration

No DLTs were observed among the three patients enrolled at dose-level 1 (40 mg/m^2^).

Among the three first patients enrolled at dose-level 2 (60 mg/m^2^), one was not assessable for DLT because he received the wrong dose (40 mg/m^2^); he was subsequently replaced by a fourth patient. This patient experienced DLT (Grade 3 neuropathy). Three additional patients were thus enrolled at the same dose-level; none of them experienced DLT.

Three patients were enrolled at dose-level 3 (70 mg/m^2^). One of them was not assessable for DLT because he received only two injections of paclitaxel due to rapid disease progression with intestinal occlusion leading to death. A fourth patient was then enrolled. None of these patients experienced DLT.

Three patients were enrolled at dose-level 4 (75 mg/m^2^). One of them experienced DLT: febrile neutropenia. Furthermore, this patient affected by cholangiocarcinoma died from disease progression immediately after the occurrence of DLT. A fourth patient was then enrolled; this patient also experienced a DLT (leucopenia not allowing administration of paclitaxel at Day 8).

Consequently, the dose escalation was stopped, and the recommended phase II dose was defined as dose-level 3 (70 mg/m^2^).

Ten additional patients were then enrolled at the recommended phase II dose. One of them experienced DLT (leucopenia not allowing administration of paclitaxel at Day 15). Considering the 13 patients treated at the recommended phase II dose and evaluable for DLT assessment, the probability of DLT is estimated at 8% (95%CI: 0.2 to 36%).

### Safety and feasibility

**Fig. 1:**
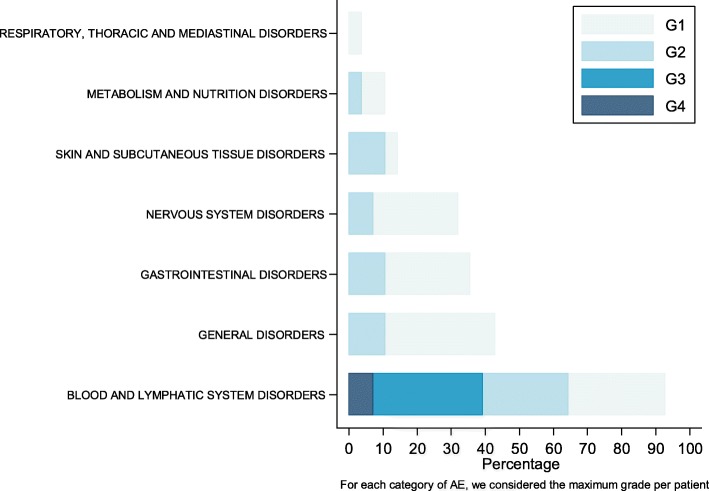
Distribution of treatment-related adverse events during the first treatment cycle (all patients, *N* = 28)

Figure 1 illustrates the distribution of grades of drug-related adverse events (AE) occurring during the 1st cycle. Overall (*n* = 28), over the first cycle, the maximum grade of drug-related AE was Grade 1 in six patients, Grade 2 in 13 patients, Grade 3 in two patients and Grade 4 in 2 patients (no AE in 5 patients). At the recommended phase II dose (*n* = 14), the maximum grade of treatment-related AE was Grade 1 in three patients, Grade 2 in 6 patients, Grade 3 in 1 patient and Grade 4 in 1 patient (no AE in three patients).

Table 2 details the distribution of the maximum grades of drug-related AE reported over the entire treatment duration per toxicity type.

**Table 2:** Drug-related adverse events reported during the entire treatment period (All patients, *N* = 28)

AE category	G 0	G 1	G 2	G 3	G 4	Total G ≥ 1		Total G ≥ 3	
Blood And Lymphatic System Disorders	0	4	9	12	3	28	100.00%	15	53.57%
Anemia	24	1	3	0	0	4	14.30%	0	0.00%
Platelet Count Decreased	27	0	0	1	0	1	3.60%	1	3.60%
Neutropenia	16	3	6	3	0	12	43.86%	3	10.71%
Febrile Neutropenia	26	0	0	1	1	2	7.10%	2	7.10%
Lymphocyte Count Decreased	0	6	10	10	2	28	100.00%	12	43.86%
Gastrointestinal Disorders	15	7	6	0	0	13	46.40%	0	0.00%
Abdominal Pain	26	1	1	0	0	2	7.10%	0	0.00%
Diarrhea	23	2	3	0	0	5	17.90%	0	0.00%
Nausea	21	5	2	0	0	7	25.00%	0	0.00%
Stomatitis	27	1	0	0	0	1	3.60%	0	0.00%
Vomiting	26	2	0	0	0	2	7.10%	0	0.00%
General Disorders	13	7	6	2	0	15	53.60%	2	7.10%
Fatigue	13	7	6	2	0	15	53.60%	2	7.10%
Metabolism And Nutrition Disorders	23	3	2	0	0	5	17.90%	0	0.00%
Anorexia	25	2	1	0	0	3	10.70%	0	0.00%
Hypoalbuminemia	27	0	1	0	0	1	3.60%	0	0.00%
Weight Loss	27	1	0	0	0	1	3.60%	0	0.00%
Nervous System Disorders	15	9	3	1	0	13	46.43%	1	3.60%
Dizziness	27	0	1	0	0	1	3.60%	0	0.00%
Dysgeusia	26	2	0	0	0	2	7.10%	0	0.00%
Peripheral Sensory/Motor Neuropathy^*^	16	8	3	1	0	12	43.86%	1	3.60%
Renal And Urinary Disorders	26	1	1	0	0	2	7.10%	0	0.00%
Hematuria	26	1	1	0	0	2	7.10%	0	0.00%
Respiratory, Thoracic And Mediastinal Disorders	24	3	1	0	0	4	14.30%	0	0.00%
Dyspnea	26	1	1	0	0	2	7.10%	0	0.00%
Epistaxis	26	2	0	0	0	2	7.10%	0	0.00%
Skin And Subcutaneous Tissue Disorders	14	7	7	0	0	14	50.00%	0	0.00%
Alopecia	14	7	7	0	0	14	50.00%	0	0.00%
Dry Skin	26	2	0	0	0	2	7.10%	0	0.00%

G 0: no AE; G 1: Grade 1 AE, G 2: Grade 2 AE, G 3: Grade 3 AE, G 4: Grade 4 AE, G 5: lethal AE

For each category type, we considered the maximum grade per patient observed over the entire treatment duration

All adverse events, classified as drug-related or not, are summarized in (Additional file 1: Table S1)

^*^Myalgia has been pooled with peripheral sensory neuropathy because this symptom reflects more a peripheral neurotoxicity than a musculoskeletal disorder in the study setting

The most frequent adverse events were hematological toxicities (28 patients, 100%); however, febrile neutropenia occurred in only two patients. Peripheral sensory/ motor neuropathy was reported in 12 patients (44%) during first cycle (8 Grade 1, 3 Grade 2 and 1 Grade 3).

Over the 1st cycle, the relative dose-intensity was > 75% for both drugs in 23/28 patients (82%). Two patients (7%) required transient treatment interruption classified as DLT. Treatment was definitively stopped for 2 other patients (7%, 1 DLT and 1 early progression), and another patient received a reduced dose by error. Five patients definitively stopped the study treatment (at least one of the drugs) after 1 cycle, and 15 stopped after 2 cycles, whereas 8 patients received more than 2 cycles of the combination (maximum, 5 cycles). The reasons for stopping the treatment were toxicity for 4 patients, progression for 21, patient’s choice for 1, physician’s decision for 1, and unknown for 1 patient.

We have observed Grade 3 lymphopenia in 12 patients. The median duration of this grade 3 lymphopenia was 2,6 months (range, 0,3-10,2). We have observed three infectious episodes in three patients: urinary tract infection, skin infection and febrile neutropenia.

### Anti-tumor activity

Table 3 depicts the activity endpoints. At the date of the analysis, all patients had progressed, with a median progression-free survival of 2.1 months (95%-CI: 1.6–3.7) in the entire population and 2.9 months (95%-CI: 1.5–5.1) at the recommended phase II dose. Two patients were still alive at 41.2 and 37.2 months after study entry, whereas 26 patients died (all from disease progression), leading to a median overall survival of 8.2 months (95%-CI: 5.1–11.7) in the entire study population and 6.8 months (95%-CI: 3.7–11.1) at the recommended phase II dose (Table 3). Growth Modulation index (GMI) was assessable in 27 patients. The median GMI was 0.7 (range, 0–3,5). GMI was ≥1.33 in 7/27 (26.0, 95%-CI: 11.0–46.0). Details on 2 patients with lung adenocarcinoma are provided in (Additional file 1: Table S2).

**Table 3:** Main efficacy outcomes overall and at the recommended phase II dose

	Recommended phase II dose			Entire study cohort		
	N	%	95% CI	N	%	95% CI
Objective response at 2 cycles	1/14	7%	0–34%	2/28	7%	1–24%
Non-progression at 2 cycles	8/14	57%	29–82%	12/28	43%	24–63%
Growth modulation index≥1.3	4/14	29%	8–58%	7/27	26%	11–46%
Median progression-free survival (months)	*N* = 14	2.9 m	1.5–5.1	*N* = 28	2.1 m	1.6–3.7
Median overall survival (months)	*N* = 14	6.8 m	3.7–11.1	*N* = 28	8.2 m	5.1–11.7

## Discussion

The key-findings of this dose-finding phase I trial are (i) the recommended phase II dose of weekly paclitaxel is 70/mg/m^2^ when administered in combination with 50 mg OMC twice a day, (ii) DLTs were mainly hematological, (iii) this combination appeared well tolerated, and (iv) objective responses were noted in patients with heavily pretreated lung adenocarcinoma.

The tolerance of the combination was mostly manageable without unexpected toxicity. The observed toxicity was as expected in terms of the nature and severity of these events. In this study, the addition of metronomic cyclophosphamide did not allow a dose escalation of weekly paclitaxel beyond 75 mg/m^2^.

The activity and safety of weekly paclitaxel as a single agent have been assessed in several phase II trials [10, 13, 14, 16–27]. In most cases, the administered dose was 80 mg/m^2^ [13, 14, 16–19, 21, 22], and doses of 90 mg/m^2^ [10] or 100 mg/m^2^ [20] are rarely reported. The objective response rate ranged from 8% [17, 22] to 38% [16]. The median progression-free survival was approximately 4 months [20]. The median overall survival ranged from 3.5 months [21] to 14.5 months [20]. The reported toxicity includes mainly hematological toxicity [16, 17, 19, 21, 22] and neuropathy [16–18, 20, 22]. In the present study, we observed two partial responses occurring in two patients with lung adenocarcinoma. This finding is consistent with the literature data that supports the activity of weekly paclitaxel in lung cancer patients [12, 13, 24].

The study had some limitations. The dose of metronomic cyclophosphamide (50 mg twice a day) could be discussed since some prior trials are based on 50–100 mg once a day. Five patients aged between 66 and 67 had been enrolled (inclusion criteria was up to 65), however regarding their very good shape, the study coordinator had provided waiver. We did not conduct any translational study to evaluate biomarkers associated with tumor response. At the time of this study, analysis of ALK, ROS and MET mutations were not part of the standard of care in lung adenocarcinoma. We do not know whether the two responding patients were affected by mutated lung adenocarcinoma. Furthermore, we did not enroll patients with ovarian cancer or bladder cancer (these patients have in most cases received weekly paclitaxel before to be considered for study entry).

## Conclusions

To conclude, as previously reported [10, 13, 14, 16–27], we found that the safety profile of weekly paclitaxel associated with oral metronomic cyclophosphamide was feasible with a manageable safety profile. With the cyclophosphamide dose of 50 mg twice a day, the Phase II recommended dose of weekly paclitaxel is 70 mg/m^2^ days 1, 8 and 15 of 28-day cycles. However, in the absence of randomization and an internal comparator, we cannot establish the therapeutic role of the addition of metronomic cyclophosphamide compared with weekly paclitaxel alone.

## Additional files

Additional file 1 **Table S1.** Adverse events (treatment related or not) reported over the entire treatment duration (all patients, *N* = 28). **Table S2.** Characteristics and outcome of patient with lung cancer. (DOCX 31 kb)

## Abbreviations

AE: Adverse events
DLT: Dose-limiting toxicity
OMC: Oral metronomic cyclophosphamide
RP2D: Recommended phase II dose
wP: weekly paclitaxel
