# Supplementary material for: Once weekly paclitaxel associated with a fixed dose of oral metronomic cyclophosphamide: a dose-finding phase 1 trial
Source: BMC Cancer. 2018 Jul 31;18:775. doi: 10.1186/s12885-018-4678-x (PMC6069824; doi:10.1186/s12885-018-4678-x)
Supplement: Supplementary file 1 — Table S1. Adverse events (treatment related or not) reported over the entire treatment duration (all patients, N = 28). Table S2. Characteristics and outcome of patient with lung cancer. (DOCX 31 kb) [file 12885_2018_4678_MOESM1_ESM.docx]

Additional file 1

Table S1- Adverse events (treatment related or not) reported over the entire treatment duration (all patients, N=28)

| **AE category** | **G 0** | **G 1** | **G 2** | **G 3** | **G 4** | **G 5** | **Total G≥1** | | **Total G ≥3** | |
| --- | --- | --- | --- | --- | --- | --- | --- | --- | --- | --- |
| **Blood And Lymphatic System Disorders** | **0** | **4** | **8** | **13** | **3** | **0** | **28** | **100%** | **16** | **57%** |
| Anemia | 5 | 19 | 3 | 1 | 0 | 0 | 23 | 82% | 1 | 4% |
| Platelet Count Decreased | 23 | 4 | 0 | 1 | 0 | 0 | 5 | 18% | 1 | 4% |
| Lymphocyte Count Decreased | 0 | 6 | 10 | 10 | 2 | 0 | 28 | 100% | 12 | 43% |
| Neutropenia | 16 | 3 | 6 | 3 | 0 | 0 | 12 | 43% | 3 | 11% |
| Febrile Neutropenia | 26 | 0 | 0 | 1 | 1 | 0 | 2 | 7% | 2 | 7% |
|  |  |  |  |  |  |  |  |  |  |  |
| **Cardiac Disorders** | **26** | **2** | **0** | **0** | **0** | **0** | **2** | **7%** | **0** | **0%** |
| Palpitations | 27 | 1 | 0 | 0 | 0 | 0 | 1 | 4% | 0 | 0% |
| Sinus Bradycardia | 27 | 1 | 0 | 0 | 0 | 0 | 1 | 4% | 0 | 0% |
|  |  |  |  |  |  |  |  |  |  |  |
| **Gastrointestinal Disorders** | **9** | **7** | **9** | **2** | **0** | **1*** | **19** | **68%** | **3** | **11%** |
| Abdominal Pain | 20 | 2 | 5 | 1 | 0 | 0 | 8 | 29% | 1 | 4% |
| Constipation | 24 | 2 | 1 | 1 | 0 | 0 | 4 | 14% | 1 | 4% |
| Diarrhea | 23 | 2 | 3 | 0 | 0 | 0 | 5 | 18% | 0 | 0% |
| Dysphagia | 27 | 1 | 0 | 0 | 0 | 0 | 1 | 4% | 0 | 0% |
| Gastric Hemorrhage | 27 | 1 | 0 | 0 | 0 | 0 | 1 | 4% | 0 | 0% |
| Nausea | 18 | 6 | 3 | 1 | 0 | 0 | 10 | 36% | 1 | 4% |
| Occlusion | 26 | 0 | 1 | 0 | 0 | 1* | 2 | 8% | 1 | 4% |
| Stomatitis | 27 | 1 | 0 | 0 | 0 | 0 | 1 | 4% | 0 | 0% |
| Vomiting | 24 | 1 | 1 | 2 | 0 | 0 | 4 | 14% | 2 | 7% |
|  |  |  |  |  |  |  |  |  |  |  |
| **General Disorders** | **7** | **7** | **10** | **3** | **1** | **0** | **21** | **75%** | **4** | **14%** |
| Chills | 26 | 2 | 0 | 0 | 0 | 0 | 2 | 7% | 0 | 0% |
| Edema Limbs | 27 | 0 | 1 | 0 | 0 | 0 | 1 | 4% | 0 | 0% |
| Fatigue | 7 | 8 | 10 | 3 | 0 | 0 | 21 | 75% | 3 | 11% |
| Fever | 22 | 5 | 1 | 0 | 0 | 0 | 6 | 21% | 0 | 0% |
| General Physical Health Deterioration | 27 | 0 | 0 | 0 | 1 | 0 | 1 | 4% | 1 | 4% |
| Retrosternal Pain | 27 | 1 | 0 | 0 | 0 | 0 | 1 | 4% | 0 | 0% |
|  |  |  |  |  |  |  |  |  |  |  |
| **Hepatobiliary Disorders** | **12** | **10** | **3** | **2** | **0** | **1**** | **16** | **57%** | **3** | **11%** |
| Alkaline Phosphatase Increased | 18 | 7 | 2 | 1 | 0 | 0 | 10 | 36% | 1 | 4% |
| Alt Increased | 18 | 8 | 1 | 1 | 0 | 0 | 10 | 36% | 1 | 4% |
| Ast Increased | 19 | 9 | 0 | 0 | 0 | 0 | 9 | 32% | 0 | 0% |
| Blood Bilirubin Increased | 21 | 4 | 2 | 1 | 0 | 0 | 7 | 25% | 1 | 4% |
| Hepatic Failure | 27 | 0 | 0 | 0 | 0 | 1** | 1 | 4% | 1 | 4% |
|  |  |  |  |  |  |  |  |  |  |  |
| **Infections And Infestations** | **25** | **0** | **3** | **0** | **0** | **0** | **3** | **11%** | **0** | **0%** |
| Skin Infection | 27 | 0 | 1 | 0 | 0 | 0 | 1 | 4% | 0 | 0% |
| Urinary Tract Infection | 26 | 0 | 2 | 0 | 0 | 0 | 2 | 7% | 0 | 0% |
|  |  |  |  |  |  |  |  |  |  |  |
| **Injury, Poisoning And Procedural Complications** | **27** | **0** | **0** | **0** | **1** | **0** | **1** | **4%** | **1** | **4%** |
| Fracture | 27 | 0 | 0 | 0 | 1 | 0 | 1 | 4% | 1 | 4% |
|  |  |  |  |  |  |  |  |  |  |  |
| **Metabolism And Nutrition Disorders** | **6** | **13** | **6** | **3** | **0** | **0** | **22** | **79%** | **3** | **11%** |
| Anorexia | 20 | 3 | 3 | 2 | 0 | 0 | 8 | 29% | 2 | 7% |
| Dehydration | 27 | 0 | 0 | 1 | 0 | 0 | 1 | 4% | 1 | 4% |
| Hyperkalemia | 26 | 1 | 1 | 0 | 0 | 0 | 2 | 7% | 0 | 0% |
| Hypoalbuminemia | 14 | 8 | 5 | 1 | 0 | 0 | 14 | 50% | 1 | 4% |
| Hypocalcemia | 25 | 2 | 1 | 0 | 0 | 0 | 3 | 11% | 0 | 0% |
| Hypophosphatemia | 25 | 3 | 0 | 0 | 0 | 0 | 3 | 11% | 0 | 0% |
| Weight Loss | 27 | 1 | 0 | 0 | 0 | 0 | 1 | 4% | 0 | 0% |
|  |  |  |  |  |  |  |  |  |  |  |
| **Musculoskeletal And Connective Tissue Disorders** | **25** | **1** | **2** | **0** | **0** | **0** | **3** | **11%** | **0** | **0%** |
| Back Pain | 26 | 0 | 2 | 0 | 0 | 0 | 2 | 7% | 0 | 0% |
| Wryneck | 27 | 1 | 0 | 0 | 0 | 0 | 1 | 4% | 0 | 0% |
|  |  |  |  |  |  |  |  |  |  |  |
| **Nervous System Disorders** | **12** | **12** | **3** | **1** | **0** | **0** | **16** | **57%** | **1** | **4%** |
| Dizziness | 26 | 1 | 1 | 0 | 0 | 0 | 2 | 7% | 0 | 0% |
| Dysgeusia | 26 | 2 | 0 | 0 | 0 | 0 | 2 | 7% | 0 | 0% |
| Dysphasia | 27 | 1 | 0 | 0 | 0 | 0 | 1 | 4% | 0 | 0% |
| Headache | 26 | 2 | 0 | 0 | 0 | 0 | 2 | 7% | 0 | 0% |
| Peripheral Sensory/Motor Neuropathy | 16 | 8 | 3 | 1 | 0 | 0 | 12 | 43% | 1 | 4% |
|  |  |  |  |  |  |  |  |  |  |  |
| **Psychiatric Disorders** | **26** | **1** | **1** | **0** | **0** | **0** | **2** | **7%** | **0** | **0%** |
| Anxiety | 27 | 1 | 0 | 0 | 0 | 0 | 1 | 4% | 0 | 0% |
| Insomnia | 27 | 0 | 1 | 0 | 0 | 0 | 1 | 4% | 0 | 0% |
|  |  |  |  |  |  |  |  |  |  |  |
| **Renal And Urinary Disorders** | **20** | **7** | **1** | **0** | **0** | **0** | **8** | **29%** | **0** | **0%** |
| Creatinine Increased | 21 | 7 | 0 | 0 | 0 | 0 | 7 | 25% | 0 | 0% |
| Hematuria | 26 | 1 | 1 | 0 | 0 | 0 | 2 | 7% | 0 | 0% |
|  |  |  |  |  |  |  |  |  |  |  |
| **Respiratory, Thoracic And Mediastinal Disorders** | **18** | **5** | **4** | **1** | **0** | **0** | **10** | **36%** | **1** | **4%** |
| Bronchial Obstruction | 27 | 0 | 1 | 0 | 0 | 0 | 1 | 4% | 0 | 0% |
| Cough | 24 | 3 | 1 | 0 | 0 | 0 | 4 | 14% | 0 | 0% |
| Dyspnea | 21 | 3 | 3 | 1 | 0 | 0 | 7 | 25% | 1 | 4% |
| Epistaxis | 26 | 2 | 0 | 0 | 0 | 0 | 2 | 7% | 0 | 0% |
| Hemoptysis | 27 | 1 | 0 | 0 | 0 | 0 | 1 | 4% | 0 | 0% |
| Nasal Congestion | 27 | 1 | 0 | 0 | 0 | 0 | 1 | 4% | 0 | 0% |
|  |  |  |  |  |  |  |  |  |  |  |
| **Skin And Subcutaneous Tissue Disorders** | **14** | **7** | **7** | **0** | **0** | **0** | **14** | **50%** | **0** | **0%** |
| Acneiform Rash | 27 | 1 | 0 | 0 | 0 | 0 | 1 | 4% | 0 | 0% |
| Alopecia | 14 | 7 | 7 | 0 | 0 | 0 | 14 | 50% | 0 | 0% |
| Dry Skin | 25 | 3 | 0 | 0 | 0 | 0 | 3 | 11% | 0 | 0% |
| Erythema Multiforme | 27 | 1 | 0 | 0 | 0 | 0 | 1 | 4% | 0 | 0% |
| Palmar-Plantar Erythrodysesthesia Syndrome | 27 | 1 | 0 | 0 | 0 | 0 | 1 | 4% | 0 | 0% |
|  |  |  |  |  |  |  |  |  |  |  |
| **Vascular Disorders** | **25** | **3** | **0** | **0** | **0** | **0** | **3** | **11%** | **0** | **0%** |
| Hot Flashes | 25 | 3 | 0 | 0 | 0 | 0 | 3 | 11% | 0 | 0% |

G 0: no AE; G 1: Grade 1 AE, G 2: Grade 2 AE, G 3: Grade 3 AE, G 4: Grade 4 AE, G 5: lethal AE.

For each category type, we considered the maximum grade per patient observed over the entire treatment duration.

* Patient at dose-level 3 not assessable for DLT due to the early stop of treatment given intestinal occlusion leading to death.

** Patient at dose-level 4 treated for a cholangiocarcinoma experienced a DLT (febrile neutropenia) and died immediately after the occurrence of DLT due to disease progression with hepatic failure.

Table S2 – Characteristics and outcome of patient with lung cancer

| Patient | 5 | 22 |
| --- | --- | --- |
| Gender | Woman | Man |
| Age | 52 | 50 |
| Histology | Adenocarcinoma | Adenocarcinoma |
| 1^st^ line: treatment, duration, best response | Cisplatin/pemetrexed, 9 weeks, Progressive disease | Cisplatin/pemetrexed/bevacizumab, 9 weeks, Progressive disease |
| 2^nd^ line: treatment, duration, best response | Erlotinib, 28 weeks, Progressive disease | Cisplatin/Docetaxel, 12 weeks, disease progression |
| 3^rd^ line: treatment, duration, best response | Docetaxel, 18 weeks, Partial response | Erlotinib, 12 weeks, disease progression |
| 4rd line: treatment, duration, best response | No | Vinorelbine, 12 weeks, disease progression |
| Dose-level in the present trial | 2 (paclitaxel 60 mg/m²°) | 5 (paclitaxel 70 mg/m²° |
| Metastasis sites | Pleura and lung | Pleura and lung |
| Best response | Partial response | Partial response |
| Treatment duration | Cyclophosphamide, 15 weeks  Paclitaxel, 15 weeks | Cyclophosphamide; 14 weeks  Paclitaxel, 6 weeks |
| Time to progression | 84 weeks | Not assessable |
| Overall survival | 168 weeks | 72 weeks |
